# Understanding the Link between Household Food Insecurity and Self-Rated Oral Health in Ghana

**DOI:** 10.3390/ijerph20010121

**Published:** 2022-12-22

**Authors:** Daniel Amoak, Joseph Asumah Braimah, Williams Agyemang-Duah, Nancy Osei Kye, Florence Wullo Anfaara, Yujiro Sano, Roger Antabe

**Affiliations:** 1 Department of Geography and Environment, Western University, London, ON N6A 5C2, Canada; 2 Department of Health and Society, University of Toronto Scarborough, Scarborough, ON M1C 1A4, Canada; 3 Department of Geography and Planning, Queen’s University, Kingston, ON K7L 3N6, Canada; 4 Department of Gender, Sexuality, and Women’s Studies, Western University, London, ON N6A 5B8, Canada; 5 Department of Sociology and Anthropology, Nipissing University, North Bay, ON P1B 8L7, Canada

**Keywords:** food insecurity, self-rated oral health, social determinants of oral health, older adults, Ghana

## Abstract

There is increasing scholarly attention on the role of food insecurity on the health of older adults in sub-Saharan Africa, including Ghana. Yet, we know very little about the association between food insecurity and self-rated oral health. To address this void in the literature, this study uses a representative survey of adults aged 60 or older from three regions in Ghana to examine whether respondents who experienced household food insecurity rated their oral health as poor compared to their counterparts who did not. We found that 34% of respondents rated their oral health as poor, while 7%, 21%, and 36% experienced mild, moderate, and severe food insecurity, respectively. Moreover, the results from the logistic regression analysis showed that older adults who experienced mild (OR = 1.66, *p* < 0.05), moderate (OR = 2.06, *p* < 0.01), and severe (OR = 2.71, *p* < 0.01) food insecurity were more likely to self-rate their oral health as poor, compared to those who did not experience any type of food insecurity. Based on these findings, we discuss several implications for policymakers and directions for future research.

## 1. Introduction

Food insecurity—defined as the limitation or uncertain availability of nutritionally adequate and safe foods or the limited or uncertain ability to acquire food in socially acceptable ways—is a persistent issue across the globe, disproportionately affecting sub-Saharan Africa (SSA), where 66% of the population experienced food insecurity in 2020 [1,2]. Research highlights that older adults have been particularly exposed to higher risks of experiencing food insecurity in SSA compared to their younger counterparts. For example, based on a pooled sample of SSA countries, Wambogo et al. [3] found that adults aged 50 or older were significantly more likely to experience severe food insecurity, compared to those younger than 50. Based on a systematic review of 22 studies, Saha and colleagues [4] also suggest that food insecurity in SSA is a critical challenge among older adults, with a specific emphasis on intra-regional variations, pointing to a wide range of prevalence of food insecurity within SSA—from 6.0% in Madagascar to 87.3% in South Sudan. In the case of Ghana, there are very few studies that document the prevalence of food insecurity among older people. Based on the sample of adults aged 50 or older, for example, Gyasi et al. [5] revealed that 44% and 9% of respondents were exposed to moderate and severe food insecurity, respectively. Blankson and Hall [6] also found that among 59 rural women aged 60 to 92, 42% and 20% missed a meal the day before and went to bed hungry, respectively.

These estimates from SSA and Ghana, in particular, may serve as unique barriers to healthy aging, as research shows that food insecurity can interact with aging-specific changes in physiological functions to negatively impact physical health conditions. Indeed, it has been found among older adults that food insecurity is associated with hypertension, cardiovascular diseases, diabetes, and functional limitations [4,7,8]. Beyond physical health, the literature shows that several indicators of mental and cognitive health, such as psychological disorders, sleep quality, depression, and working memory are adversely impacted by food insecurity [9,10,11,12].

Although these studies are useful, existing studies largely overlook the potential role of food insecurity experienced by older people in a resource-limited context of SSA, including Ghana, on oral health, defined as a state of being free from chronic mouth and facial pain, oral and throat cancer, oral infection and sores, periodontal disease, tooth decay, tooth loss, and other diseases and disorders that limit an individual’s capacity in biting, chewing, smiling, speaking, and psychosocial well-being [13]. In 2017, 400 million people suffered from oral health problems in SSA, thereby receiving increasing global attention as a critical public health concern in the region. Research also shows that oral health issues have been particularly concerning among older adults in Ghana where the prevalence of caries and periodontitis is higher among those aged 55 years or older [14]. These dental problems often share modifiable risk factors with other common non-communicable diseases, which may include smoking, drinking alcohol, and engaging in adequate levels of exercise [15]. In addition, certain oral health conditions, such as periodontal diseases are found to be systemically linked to other chronic diseases, such as hypertension and diabetes, especially among older adults [16].

With the increasing recognition of oral health issues coupled with the high prevalence of food insecurity, there is an urgent need to examine whether the negative health impacts of food insecurity can be extended to oral health among older adults in the context of Ghana and other SSA countries. In this context, the current study uses a representative survey of adults aged 60 or older from three regions of Ghana to explore whether (1) people rated their oral health differently based on their household food security status, and if so, (2) the relationship between household food security status and self-rated oral health is modified after accounting for theoretically relevant variables.

## 2. Materials and Methods

### 2.1. Data

**Figure 1:**
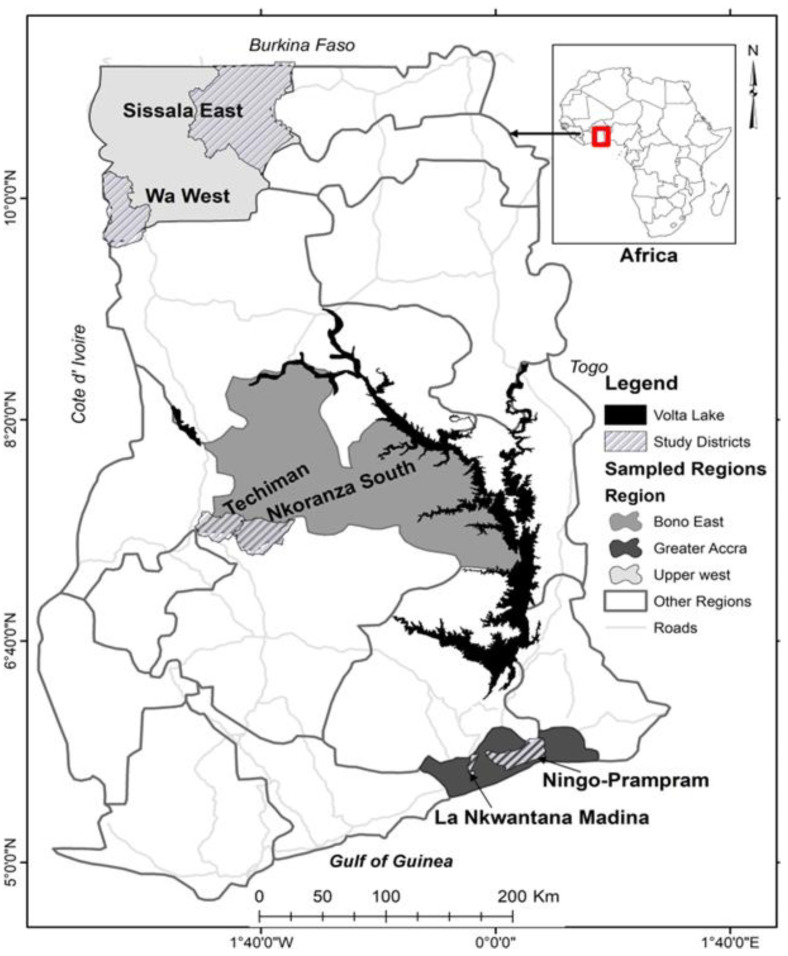
Map of Ghana showing the study regions.

This study used data from a cross-sectional survey of older adults (aged 60 years and over) in Ghana. Data collection was done from June to August 2019 with the assistance of experienced research assistants from the University of Ghana, the University for Development Studies, and the Catholic University College, Ghana. Research assistants were trained on the survey and various ethical issues and were each made to sign a confidentiality agreement prior to the data collection. Ethical approval for the study was obtained from the Queen’s University General Research Ethics Board. The survey was administered in three purposively selected regions, including the Upper West Region, Bono East Region, and Greater Accra Region. See Figure 1.

The Upper West Region is considered the poorest region in Ghana, and the rest of northern Ghana is characterised by a high incidence of poverty, food insecurity, and worsening climatic conditions [17,18]. In contrast, the Bono East Region, considered the ‘breadbasket’ of Ghana, has favourable rainfall patterns and good soils for the cultivation of food and cash crops. These factors, coupled with the region’s relative proximity to longstanding migrant networks, make it a preferred destination for migrants, typically from northern Ghana, including the UWR, who wish to work in the agricultural sectors [19]. In Ghana, the Greater Accra Region is the most urbanised region, with about 92% living in urban areas (Ghana Statistical Service, 2021c). The poverty incidence is 2.5%, which is well below that of Bono East (26.8%) and the Upper West (70.9%) regions, as well as the national average of 23.4% [20]. Although these regions have different socioeconomic and demographic characteristics, research has shown that older people in these regions remain critical support pillars, especially when caring for grandchildren and other vulnerable members of their communities [21,22]. Accordingly, the inclusion of these regions can be useful for reflecting the diverse nature of older people in Ghana.

In terms of the sampling procedure, we randomly drew two districts from each region. Ten enumeration areas were then randomly sampled from each district. Finally, we identified and interviewed one respondent each from randomly chosen households within these enumeration areas. Based on information from the 2010 Population and Housing Census [18] we also constructed calibration weights for the regression analysis, which enabled us to account for the differences in regional characteristics between sample and population characteristics. In addition, due to the multi-stage sampling strategy, sampling weights were further constructed for each stage of sampling based on the probabilities of each sampling unit being selected as part of the sample. To this end, our analytical sample included 1073 older adults. Standardised data collection instruments on food security, health, health behaviours, and access to care were adapted from the Ghana Living Standards Survey [20] and the WHO Study on Global Ageing and Adult Health [23]. Informed consent to participate in the study and for publication were obtained from all participants involved in the study.

### 2.2. Dependent Variable

Respondents were asked to self-rate their oral health with five possible response categories, i.e., ‘excellent’, ‘very good’, ‘good’, ‘fair’, and ‘poor’. Existing studies commonly combine these categories into two groups, i.e., ‘good’ and ‘poor’ [24,25]. To situate our analytical approach in the context of broader literature, we adopted this coding strategy to create a dependent variable called ‘self-rated oral health’. Specifically, we combined ‘excellent’, ‘very good’, and ‘good’ into ‘good’ and ‘fair’ and ‘poor’ into ‘poor’ (0 = good; 1 = poor). Self-rated oral health has been documented as a valid and reliable indicator of overall oral health at the population level [26]. Importantly, this subjective indicator has been found to be correlated with clinical assessments of oral health, including dental caries and pain, periodontal diseases, as well as the decayed, missing, and filled teeth index [27].

### 2.3. Independent Variable

Food security status is a focal explanatory variable, which is constructed using the nine-item Household Food Insecurity Access Scale (HFIAS) developed by Coates et al. [28]. Respondents were asked to answer questions about the frequency of occurrence of each food insecurity situation on a Likert scale (0 = rarely; 1 = sometimes; 2 = often) when they experienced each situation. Based on the combination of responses from nine questions, respondents can be categorised into four groups, i.e., food secure, mildly insecure, moderately insecure, and severely insecure (0 = food secure; 1 = mildly insecure; 2 = moderately insecure; 3 = severely insecure).

### 2.4. Control Variables

We used the social determinants of health framework as a conceptual map to introduce control variables. This framework indicates that health is a function of the complex interplay of two broad factors, namely intermediary and structural factors [29]. Informed by this framework and other empirical studies [30,31], we introduce control variables that reflect these factors. Specifically, intermediary factors include the region of residence (0 = Upper West; 1 = Bono East; 2 = Greater Accra), place of residence (0 = urban; 1 = rural), physical activities (0 = yes; 1 = no), alcohol consumption (0 = no; 1 = yes), tobacco consumption (0 = no; 1 = yes), and age of respondents (0 = ≥80; 1 = 70–79; 2 = 60–69). We further add structural factors, such as household size (0 = ≤5; 1 = 10–6; 2 = ≥11), group participation (0 = yes; 1 = no), religion (0 = Christian; 1 = Muslim; 2 = traditionalist; 3 = no religion), marital status (0 = divorced; 1 = married; 2 = single; 3 = widowed), gender (0 = male; 1 = female), education (0 = higher education; 1 = secondary education; 2 = primary education; 3 = no education), occupation (0 = civil servant; 1 = agriculture; 2 = business; 3 = unemployed; 4 = other), and household wealth (0 = rich; 1 = middle; 2 = poor). The household wealth variable was compiled using an asset index adapted from the Demographic and Health Survey, which was computed using the principal component analysis.

### 2.5. Statistical Analysis

There are two separate analyses for this study. First, we employed a univariate analysis to understand the characteristics of the analytical sample. Second, a regression analysis was applied to understand the relationship between household food security status and self-rated oral health. Due to the binary nature of the dependent variable, we relied on the logistic regression analysis. We applied sampling weights to account for the hierarchical nature of the data structure. Models were built sequentially. Model 1 explored the bivariate association between food security status and self-rated oral health, while Models 2 and 3 further added intermediary and structural factors, respectively.

## 3. Results

Table 1 shows sample characteristics. We found that 34% of respondents rated their oral health as poor. For food security status, 36%, 21%, and 7% experienced severe, moderate, and mild food insecurity, respectively, while only 36% were in the food secure category. About half of the respondents (49%) were from urban areas. In terms of health-related behaviours, 36% and 18% engaged in the regular consumption of alcohol and tobacco products, respectively. The largest age group was 60–69 (58%), followed by 70–79 (24%), and those aged 80 or older (18%). Slightly more than half of the respondents participated in social groups (55%) and were married (56%) and male (53%). The largest religious group was Christian (61%), followed by Muslim (26%) and traditionalist (9%). It is also noteworthy that only 30% had secondary education or higher. The largest occupational group was agriculture (35%), followed by business (33%) and those who were unemployed (13%).

Table 2 shows the results from the logistic regression analysis. In Model 1, we find at the bivariate level that people who experienced severe (OR = 3.06, *p* < 0.01), moderate (OR = 2.61, *p* < 0.01), or mild (OR = 1.53, *p* < 0.1) food insecurity were more likely to rate their oral health as poor, compared to those who did not experience food insecurity. After we included intermediary factors in Model 2, it is interesting that the bivariate relationship between food security status and self-rated oral health observed in Model 1 remained largely consistent. Specifically, compared to those who did not experience food insecurity, people who experienced severe (OR = 2.36, *p* < 0.01), moderate (OR = 1.62, *p* < 0.01), or mild (OR = 1.60, *p* < 0.1) food insecurity were more likely to rate their oral health as poor. This relationship did not drastically change in Model 3 when we further accounted for structural factors. In Model 3, it was found that the odds of participants rating their oral health as poor were higher among those who experienced severe (OR = 2.05, *p* < 0.01), moderate (OR = 1.64, *p* < 0.01), or mild (OR = 1.66, *p* < 0.05) food insecurity, compared to those who did not experience food insecurity.

In addition to food security status, we found that intermediary and structural factors were significantly associated with self-rated oral health in a fully adjusted model (see Model 3). For example, respondents from Bono East were less likely to rate their oral health as poor compared to those from the Upper West (OR = 0.52, *p* < 0.01). By contrast, compared to those from urban areas, respondents from rural areas were more likely to rate their oral health as poor (OR = 1.40, *p* < 0.01). In addition, respondents who did not engage in regular physical activities were more likely to rate their oral health as poor compared to those who did (OR = 1.41, *p* < 0.01). Similarly, respondents who regularly consumed tobacco products were more likely to rate their oral health as poor compared to those who did not (OR = 1.36, *p* < 0.05). In terms of age, those aged 70–79 (OR = 0.62, *p* < 0.05) and 60–69 (OR = 0.66, *p* < 0.01) were less likely to rate their oral health as poor compared to their counterparts aged 80 or older. We also found that respondents who did not participate in social groups were more likely to rate their oral health as poor compared to those who did (OR = 1.34, *p* < 0.05). Compared to their Christian counterparts, traditionalist respondents were also more likely to rate their oral health as poor (OR = 1.40, *p* < 0.1). For socioeconomic status, respondents without any formal education (OR = 1.88, *p* < 0.05) and those from poor households (OR = 1.61, *p* < 0.01) were more likely to rate their oral health as poor compared to their more educated and wealthier counterparts.

## 4. Discussion

There is increasing attention in the literature on the role of food insecurity on the health of older adults in SSA, including Ghana. Indeed, evidence points to food insecurity as a critical risk factor for poor physical, mental, and cognitive health. Using a representative survey of people aged 60 or older from three regions of Ghana, the current study aims to advance the literature by examining whether food security status is associated with self-rated oral health among older adults.

We found that older adults who experienced household food insecurity—regardless of its severity, i.e., mild, moderate, or severe food insecurity—were more likely to report their oral health as poor, compared to those without it, even after accounting for intermediary and structural factors informed by the social determinants of the health framework. Similar findings have been reported among older adults in the United States. For example, Lee et al. [32] indicated that adults aged 60 or older with middle or severe food insecurity were more likely to have untreated tooth decay compared to their counterparts without food insecurity. In addition, it was found that food insecurity is associated with a higher prevalence of poor self-reported oral health among adults aged 50 or older [33]. Comparing our results to existing evidence, it is possible that the role of food insecurity on poor oral health can be extended to older adults in the resource-limited context of SSA, including Ghana.

There are several possible mechanisms to explain these associations between food insecurity and poor oral health. For example, research suggests that the prevalence of poor oral health, such as fewer teeth, the loss of attachment, and pocket depth are often lower among older adults with higher frequencies of consuming diverse types of food items, such as potatoes, meat, fish and shellfish, vegetables, nuts, and fruit [34,35]. In this context, older adults who experience food insecurity may face unique challenges to achieving optimal oral health due to their inability to consume food items from a wide range of nutritional groups. In addition, it has been found that food insecurity is a critical barrier to healthcare utilization, as food-insecure households are often required to prioritise other daily needs, such as food and shelter [36]. Indeed, income levels have been found to be significant determinants of healthcare-seeking behaviors among older adults in Ghana, which are often lower due to inadequate social protection [37]. Specifically, the lack of pension coverage, the irregularity of payments, as well as diminished social support from friends and family, have been linked to food insecurity among older adults [38]. Therefore, it is possible that older adults who experience food insecurity may be restricted from using dental care services, which has been documented as one of the most important predictors of positive oral health.

We also found that other control variables are significantly associated with self-rated oral health. For example, health-related behaviours such as smoking and not exercising regularly were significant predictors of poor oral health among older adults. These findings have been commonly reported in other studies in SSA [39]. In addition, our findings show that the oldest age group (80 years or older) had the greatest risk of poor oral health. This finding is consistent with previous research [40,41], arguing that the aging process can negatively affect oral health, especially in countries lacking dental care infrastructure, such as Ghana. This situation may be heightened in certain geographic contexts, such as the Upper West Region as well as rural areas, reflecting on our finding that older adults in these regions had higher rates of reporting poor oral health. Finally, our results point to the importance of social and economic resources on optimal oral health. Specifically, older adults who participated in social groups had better oral health, which may point to social capital as a critical determinant of oral health [42]. Similarly, higher socioeconomic statuses, such as higher levels of education and household wealth, were positively associated with self-rated oral health, which is consistent with previous research that explains these characteristics as unique structural resources that often help achieve better oral health at the population level [43].

Despite these usefulness of these findings, there are some limitations to this study. For example, we used a cross-sectional survey to explore the relationship between food insecurity and self-rated oral health. As we do not know the temporal order of these two events, our results are limited to statistical associations and cannot claim any causal relationships. In addition, we relied on self-rated oral health, which is a subjective indicator of oral health. Future research should integrate clinical indicators of oral health, such as the decayed, missing, and filled teeth index, untreated cavities, and salivary flow [44]. It is also noteworthy that our data did not include relevant indicators of oral health behaviours such as dental care utilization and tooth brushing, which are considered important factors associated with oral health [45]. These limitations indicate an urgent need to collect a nationally representative longitudinal survey that can comprehensively assess oral health and oral health needs among older adults in SSA, including Ghana. Despite these limitations, our study is one of a few that have explored the association between food insecurity and oral health among older adults in Ghana.

Notwithstanding the above limitations, we have several recommendations for policymakers. Food insecurity often leads to undernutrition and malnutrition among older adults, which have been documented to be important predictors of poor oral health. With this mechanism in mind, intervention programs may benefit from offering community-level food and nutrition services where household members can have access to a balanced diet, which is often made up of foods from the five food groups, namely fruits and vegetables, starchy food, dairy, protein, and fat. Moreover, food-insecure households are often required to reconsider their distributions and allocations of financial resources, as they need to choose items that are more immediately necessary for survival, such as food and housing over less immediately necessary ones, including access to dental care services. In this context, it is crucial to implement culturally appropriate dental care that is financially accessible to older adults in food-insecure households, which may help improve and maintain their oral health.

## Figures and Tables

**Table 1:** Sample characteristics.

	Percentage
Self-rated oral health	
Good	66
Poor	34
Food security status	
Food secure	36
Mildly insecure	7
Moderately insecure	21
Severely insecure	36
Region of residence	
Upper West	35
Bono East	36
Greater Accra	29
Place of residence	
Urban	49
Rural	51
Physical activities	
Yes	50
No	50
Alcohol consumption	
No	64
Yes	36
Tobacco consumption	
No	82
Yes	18
Age	
≥80	18
70–79	24
60–69	58
Household size	
≤5	40
6–10	37
≥11	23
Group participation	
Yes	55
No	45
Religion	
Christian	61
Muslim	26
Traditionalist	9
No religion	4
Marital status	
Divorced	9
Married	56
Single	7
Widowed	28
Gender	
Male	53
Female	47
Education	
Higher	16
Secondary	14
Primary	29
No education	41
Occupation	
Civil servant	12
Agriculture	35
Business	33
Unemployed	13
Other	7
Household wealth	
Rich	33
Middle	33
Poor	33
Total	1073

**Table 2:** Logit models predicting self-rated oral health.

	Model 1			Model 2			Model 3		
	OR	95% CI		OR	95% CI		OR	95% CI	
Food security status									
Food secure	1.00			1.00			1.00		
Mildly insecure	1.53 *	0.95	2.48	1.60 *	0.97	2.65	1.66 **	1.02	2.72
Moderately insecure	2.61 ***	1.91	3.56	1.62 ***	1.14	2.30	1.64 ***	1.15	2.35
Severely insecure	3.06 ***	2.32	4.02	2.36 ***	1.76	3.18	2.05 ***	1.52	2.77
Region of residence									
Upper West				1.00			1.00		
Bono East				0.34 ***	0.25	0.45	0.52 ***	0.36	0.76
Greater Accra				1.06	0.80	1.40	1.30	0.87	1.92
Place of residence									
Urban				1.00			1.00		
Rural				1.66 ***	1.32	2.09	1.40 ***	1.09	1.81
Physical activities									
Yes				1.00			1.00		
No				1.50 ***	1.18	1.89	1.41 ***	1.10	1.81
Alcohol consumption									
No				1.00			1.00		
Yes				1.30 **	1.03	1.64	1.08	0.84	1.38
Tobacco consumption									
No				1.00			1.00		
Yes				1.25 *	0.96	1.64	1.36 **	1.02	1.82
Age									
≥80				1.00			1.00		
70-79				0.56 ***	0.40	0.78	0.62 ***	0.44	0.87
60-69				0.47 ***	0.35	0.63	0.60 ***	0.44	0.83
Household size									
≤5							1.00		
10-6							1.06	0.80	1.41
≥11							1.12	0.78	1.61
Group participation									
Yes							1.00		
No							1.34 **	1.05	1.72
Religion									
Christian							1.00		
Muslim							0.96	0.71	1.30
Traditionalist							1.40 *	0.95	2.05
No religion							1.04	0.59	1.83
Marital status									
Divorced							1.00		
Married							0.79	0.52	1.19
Single							0.75	0.40	1.41
Widowed							1.06	0.69	1.62
Gender									
Male							1.00		
Female							0.97	0.74	1.26
Education									
Higher							1.00		
Secondary							1.00	0.52	1.91
Primary							1.51	0.83	2.74
No education							1.88 **	1.01	3.49
Occupation									
Civil servant							1.00		
Agriculture							1.44	0.75	2.75
Business							0.59	0.27	1.26
Unemployed							0.98	0.52	1.86
Other							1.56	0.79	3.06
Household wealth									
Rich							1.00		
Middle							1.24	0.86	1.81
Poor							1.61 ***	1.14	2.26
F	22.88 ***			17.39 ***			7.42 ***		

* *p* < 0.1, ** *p* < 0.05, *** *p* < 0.01.

## Data Availability

The data presented in this study are available on request from the corresponding author. The data are not publicly available due to restrictions by Queen’s University General Research Ethics Board.
